# Cell-free DNA methylation profiling reveals injury types and severities in swine rotational and contusional models of traumatic brain injury

**DOI:** 10.1186/s40478-026-02384-x

**Published:** 2026-07-28

**Authors:** Shih-Han Kao, Larry N. Singh, Sanjeev Chawla, Hunter A. Gaudio, Kevin D. Browne, Samuel S. Shin, Lucas J. Hobson, Sarah Kenna, Jonathan Starr, M. Katie Weeks, Nicholas J. Widmann, Anthony M. Davis, Takayuki Sueishi, Karli Wulwick, Jenna Sittineri, D. Kacy Cullen, Todd J. Kilbaugh

**Affiliations:** 1 Resuscitation Science Center, Anesthesiology & Critical Care Medicine, Children’s Hospital of Philadelphia, Philadelphia, PA USA; 2 Department of Radiology, Perelman School of Medicine at the University of Pennsylvania, Philadelphia, PA USA; 3 Center for Brain Injury & Repair, Department of Neurosurgery, University of Pennsylvania School of Medicine, Philadelphia, PA USA; 4 Center for Neurotrauma, Neurodegeneration & Restoration, Corporal Michael J. Crescenz Veterans Affairs Medical Center, Philadelphia, PA USA; 5 Department of Neurology, University of Pennsylvania School of Medicine, Philadelphia, PA USA; 6 Department of Bioengineering, University of Pennsylvania School of Engineering and Applied Science, Philadelphia, PA USA

**Keywords:** cfDNA, Methylome, TBI biomarkers

## Abstract

Early diagnosis of traumatic brain injury (TBI) is crucial to guide treatment and improve recovery. Yet neuroimaging—the current gold standard for detection—cannot detect subtle biochemical changes and is insufficient to diagnose many TBI cases. Consequently, there is an urgent need for early, brain-associated biomarkers of TBI. Following injury, brain cells release cell-free DNA (cfDNA) into peripheral blood due to neurovascular disruption. DNA methylation patterns, which inform tissue specificity, could serve as biomarkers for TBI severity and progression. To test this hypothesis, we analyzed cfDNA from peripheral blood of swine models of TBI having injuries of differing severity and type: mild and moderate contusional, and mild and moderate rotational injuries. Using whole genome bisulfite sequencing, we identified distinct brain-associated cfDNA epigenetic signatures of affected brain regions, and the underlying biological processes associated with each injury type and severity. In these brain regions, proton magnetic resonance spectroscopic imaging (^1^H MRSI) independently confirmed changes in biomarkers of brain health and function (choline and N-acetylaspartate metabolites). Droplet digital PCR validated that cfDNA differentially methylated regions (DMRs) tracked TBI progression. Our findings provide evidence that brain-associated cfDNA methylome signatures, combined with ^1^H MRSI, can characterize the neurobiological processes at sites of TBI in a preclinical model. These results support further investigation of cfDNA as a non-invasive biomarker for TBI detection and monitoring, pending validation in larger and clinically diverse cohorts.

**Supplementary Information:**

The online version contains supplementary material available at https://doi.org/10.1186/s40478-026-02384-x.

## Introduction

Traumatic brain injury (TBI) is a global cause of morbidity and mortality [1], typically occurring when a sudden impact, penetration, or rapid brain movement damages the brain. Since TBI results from many causes, symptoms and outcomes are wide-ranging, from contusions and concussions to severe, long-term disabilities [2–4]. Furthermore, TBI is increasingly linked to long-term neurobehavioral, neuropsychological impairments, and neurodegenerative conditions [5, 6] including chronic traumatic encephalopathy (CTE) [7], and Alzheimer’s disease [8, 9]. Hence, early diagnosis and treatment for TBI is essential for personalized prevention and treatment.

While neuroimaging techniques, such as CT scans and MRIs, remain the gold standard approach to diagnose TBI, they often fail to identify diffuse or minute alterations of certain brain injuries [10], resulting in potential underdiagnosis of the disease. Emerging protein biomarkers, such as neurofilament light (NFL) [11], ubiquitin C-terminal hydrolase-L1 (UCH-L1) [12], and glial fibrillary acidic protein (GFAP) [12], have improved diagnostic capabilities and continue to play an important role in TBI assessment. However, given the complexity and heterogeneity of TBI, a single class of biomarkers may not fully capture the diverse biological processes underlying injury and recovery. Therefore, new complementary approaches are urgently needed to comprehensively provide insight into the cellular and molecular mechanisms of brain injury.

A promising complementary approach involves circulating cfDNA, which consists of 120–220 bp DNA fragments released into the bloodstream. In healthy individuals, cfDNA fragments primarily originate from apoptotic hematopoietic cells [13], vascular endothelial cells, and hepatocytes [14]. Importantly, DNA methylation patterns preserve tissue-specific epigenetic information, enabling inference of the tissue of origin. In TBI, cfDNA released from injured brain cells into peripheral circulation may therefore provide insight into the location and biological state of affected brain regions. Previous studies have shown that elevated cfDNA levels are associated with severity of trauma, and posttraumatic complications [15, 16]. Building on this, cfDNA methylation profiling may offer the potential to capture the mechanistic information about the state and origin of recently damaged or dying brain cells, complementing existing protein biomarkers.

To precisely identify cfDNA originating from brain tissue, we leverage the methylation patterns of cfDNA fragments that are unique to brain tissue. DNA methylation predominantly occurs on the cytosine that precedes a guanine residue (CpG) [17] and is both a pivotal epigenetic regulator and signature for cell identity [18]. Tissue of origin of cfDNA fragments can be determined by comparing the methylation profiles of cfDNA fragments with reference methylomes from different tissues. To capture methylation profiles, whole genome bisulfite sequencing (WGBS) is the most comprehensive approach, providing single-base resolution and unbiased detection genome-wide [19], as well as enabling precise methylation identification at each cytosine.

Here we present a brain-associated cfDNA methylome from two swine TBI models at two severity levels: mild contusional injury (miCCI), moderate contusional injury (moCCI), mild rotational injury (miRAI), and moderate rotational injury (moRAI). We used cutting-edge tools to analyze brain-associated epigenetic signatures of cfDNA and identify affected brain regions and biological processes in each injury type and severity. Changes in brain metabolite levels, which are closely linked to brain injury and dysfunction, were also examined using ^1^H MRS to corroborate the findings from cfDNA analyses. This study uncovers the unique cfDNA fragments that can potentially be utilized as brain injury type- and severity-specific biomarkers.

## Materials and methods

### Animal preparation

A total of 36 pigs weighing 25–40 kg were used for this study. The animals were randomly assigned to five groups: sham, miCCI, moCCI, miRAI, and moRAI. Equal number of male and female pigs were allocated to each group. Animals were fasted overnight prior to the procedures. On the morning of surgery/injury, swine were sedated with an intramuscular injection of ketamine (20 mg/kg) and xylazine (2 mg/kg), as previously described [20] and masked with isoflurane (4% isoflurane, 2L O_2_). Animals were then intubated and maintained on 2–2.5% isoflurane in 2 L oxygen for the duration of the surgical procedure and traumatic brain injury. After intubation and under sterile conditions, each animal received a central venous catheter (CVC) via cephalic vein that terminated in the superior vena cava and was tunneled subcutaneously to exit mid-scapula. [21]. Incisions were closed with absorbable suture and protected with Tegaderm patches. Once surgery was complete and CVC patency confirmed, animals were subjected to CCI or rotational acceleration injury. Sham animals (n = 4) received only CVC and scalp incisions; however, anesthesia time was consistent with those animals undergoing TBI. Non-invasive blood pressure, heart rate, respiratory rate, oxygen saturation, and end-tidal CO_2_ were monitored throughout the experiment.

### Controlled cortical impact (CCI) injury

Under sterile conditions, a curvilinear scalp incision was made, exposing the sagittal and right coronal sutures. A 2 cm craniectomy was performed such that the center of the craniectomy was placed 1.5 cm from the coronal and sagittal sutures. This enabled exposure of the rostral gyrus and allowed a 1 cm margin around the indenter tip of the cortical impact device [20]. A stellate opening of the dura allowed access to the cortical surface and the device was stabilized against the skull with pressure screws. With a spring constant of 4 m/second, the spring-loaded tip indented the cortical rostral gyrus at two depths: (1) miCCI at 6.0–7.0 cm in depth, and (2) moCCI at 8.0–9.0 cm in depth. The device was then removed and the scalp sutured to conclude the procedure.

### Rotational acceleration injury (RAI)

Brain trauma was induced via head rotational acceleration as previously described in detail [22–24]. Briefly, the animals’ heads were secured to a padded snout clamp, which in turn was attached to a pneumatic actuator (HYGE, Inc, Kittanning, PA) that converts linear motion into angular motion (rotational acceleration). Triggered release of pressurized nitrogen drives the linkage assembly. Injury was performed in the sagittal plane at two velocities: (1) miRAI at 85–95 radians/second, and (2) moRAI at 100–110 radians/second. Magneto-hydrodynamic sensors (Applied Technology Associates, Albuquerque, NM) were used to record angular displacement; the sampling rate for the sensor was 10 kHz. Immediately prior to induction of the injury, the anesthesia tubing was disconnected from the endotracheal tube. The animals’ heads were released from the clamp following injury.

For both CCI and RAI procedures: Upon completion of the injury, the animals were transported back to their cages and given access to food and water ad libitum. Daily health checks were made to ensure that there were no adverse events during recovery. Swine were outfitted with mesh jackets for catheter protection and were singly-housed to prevent cage mates from gnawing at the catheter hub; however, each animal was able to interact with a neighboring pig and had full view of conspecifics within the colony. Catheters were flushed daily with sterile normal saline for the 14 days survival period.

### Blood collection, cfDNA extraction, and bisulfite conversion

Blood was drawn into the cf-DNA/cf-RNA Preservative Tubes (Norgen Biotek, Ontario, Canada) via CVC placed at the superior vena cava at the following times: pre-injury (pre), 2 h, 6 h, 24 h, 3 days, and 14 days after injury. Plasma was isolated by the standard dual centrifugation method. CfDNA was extracted using the NextPrep-Mag cfDNA isolation kit (PerkinElmer, Waltham, MA) and quantified using the Qubit 1 × dsDNA high sensitivity assay kit (Thermo Fisher Scientific, Waltham, MA) with the Qubit 4 Fluorometer (Thermo Fisher Scientific, Waltham, MA). The fragment size of cfDNA was examined using the cell-free DNA ScreenTape assay by the Agilent 4200 TapeStation systems (Agilent Technologies, Santa Clara, CA). CfDNA was bisulfite converted using the EZ DNA methylation-lightning kit (Zymo Research, Irvine, CA) according to the manufacturer’s protocol. Swine genomic DNA (gDNA) samples isolated from the cortex (PG-212), cerebellum (PG-202), blood (PG-705), and liver (PG-314) were purchased from Zyagen (San Diego, CA) and were also subjected to the above-mentioned bisulfite conversion before being sequenced.

### Sequencing and data processing

Libraries for WGBS were prepared from the bisulfite-converted samples using the Accel-NGS® Methyl-Seq DNA Library Kit (Integrated DNA Technologies, Coralville, IA) with Methyl-Seq Unique Dual Indexing Kit (Integrated DNA Technologies) following the manufacturer’s instructions. Libraries were quantified using Qubit 1 × dsDNA high sensitivity assay kit with the Qubit 4 fluorometer. Sequencing was performed on the NovaSeq 6000 platform (Illumina, San Diego, CA) generating 2 × 150 paired-end reads at 30 × coverage. Sequencing reads were demultiplexed, quality checked by FastQC (v0.12.1), trimmed by TrimGalore (v0.6.10), and mapped to the pig reference genome assembly (Sscrofa11.1_v100) by Bismark (v0.24.2) [25]. Bismark was also used for PCR deduplication and post-alignment quality control. Bismark methylation extraction was applied to extract the methylation call for CpGs in each sample.

### Differential methylation analysis

Differential methylation analysis was performed using the standard SeqMonk analytic workflow (https://www.bioinformatics.babraham.ac.uk/projects/seqmonk/). Methylation values were quantified by the “bisulphite methylation over features” pipeline in Seqmonk. This pipeline calculates methylation over defined genomic features/windows from sufficiently observed CpG calls using user-defined coverage and feature-observation thresholds (see Supplementary Method and Data). Importantly, this quantitation step does not assign a tissue-of-origin probability or a CpG-/DMR-level probability of brain specificity.

A common set of 25-CpG genomic windows/probes was generated using the reference brain tissues, non-brain reference tissues, and injury cfDNA samples. To construct a swine brain-associated methylation atlas, we first identified genomic windows that showed significant differential methylation between brain tissues and non-brain reference tissues. Cortex-associated DMRs were selected if methylation differed significantly between cortex and blood and between cortex and liver, with FDR < 0.001 for both comparisons. Cerebellum-associated DMRs were selected using the same criterion, requiring significant differential methylation between cerebellum and blood and between cerebellum and liver, with FDR < 0.001 for both comparisons. DMRs identified from cortex and cerebellum were combined to create the swine brain-associated methylation atlas. We then evaluated injury-associated cfDNA methylation changes within this atlas by comparing post-injury cfDNA samples with the corresponding pre-injury state. Brain-associated injury DMRs were selected if methylation differed between any post-injury time point and the pre-injury state with FDR < 0.05.

For ddPCR assay selection, brain-associated hypermethylated DMRs were defined as regions where the level of methylation in cortex or cerebellum exceeded that of blood and liver by more than 20% (differential (diff) methylation > 20%), and hypomethylated DMRs were defined as diff methylation < 20%.

We defined differentially methylated genes (DMGs) if the start and end of a DMR exactly overlapped the entire gene body of a gene. For cross-species analyses, swine DMGs were mapped to their human orthologs before enrichment testing against human brain- and blood-elevated gene sets.

### Functional enrichment analysis

We analyzed Gene Ontology (GO) enrichment of DMGs using ShinyGO (v0.80) [26]. To reduce potential contributions from hematopoietic-derived cfDNA, a human blood gene atlas was obtained from The Human Protein Atlas Blood Atlas (https://v19.proteinatlas.org/humanproteome/blood) and cross-referenced with swine orthologs. Genes with blood-associated expression signatures were then excluded from the DMG list prior to enrichment analysis. The Duroc genes Sscrofa11.1 was selected as the genome assembly. GO Biological Process and Cellular Component terms with FDR < 0.05 were considered significantly enriched. Significant GO terms were ranked first by fold enrichment, followed by FDR and the number of overlapping genes (Supplementary Method and Data).

### Brain region analysis

To analyze gene expression across brain regions for a specific injury type, RNA expression values of the DMGs in the indicated brain regions were obtained from the HPA pig brain RNA-Seq dataset on the human protein atlas website (www.proteinatlas.org). These RNA expression values of the DMGs, represented as normalized Transcripts Per Million (nTPM), were then transformed to z-scores using the mean and standard deviation of all pig brain gene-by-region nTPM values in the atlas. For each set of DMGs from each condition (mi/mo CCI/RAI), we then computed the median RNA expression z-score for each brain region across the DMGs in that set. This median value was used as a regional expression score to rank brain regions.

### Detection of DNA methylation by ddPCR

We used the ddPCR EvaGreen assay for DMR screening. Briefly, the ddPCR EvaGreen reaction mixture consisted of the 2 × EvaGreen supermix (#1,864,034, Bio-Rad, Hercules, CA), forward and reverse primer (250 nM), and 2 µl bisulfite-converted DNA in a total volume of 22 µl. The droplet was generated in the QX200 droplet generator (Bio-Rad) and the mixture was transferred to a PCR plate. The thermal cycling was carried out in a C1000 thermal cycler (Bio-Rad) and the cycling conditions were 95 °C for 5 min, followed by 40 cycles at 95 °C for 30 secs and 60 °C for 1 min with a final 5 min at 4 °C and 10 min at 90 °C for signal stabilization. The DMRs that were significantly different (Student’s* t* test, p < 0.05) between the swine cortex/cerebellum and the blood/liver were selected for further validation by the ddPCR probe assay. Swine methylated and unmethylated controls were included as the positive and negative control for the methylation-specific primer design. The ddPCR probe assay consisted of the 2 × ddPCR Supermix for probes (no dUTP, #1,863,024, Bio-Rad), forward and reverse primer (900 nM), probe (250 nM), and 2 ul bisulfite-converted DNA. The PCR cycling conditions were as follows: 95 °C for 10 min, followed by 40 cycles at 94 °C for 30 secs and 60 °C for 1 min. The droplets were counted using a QX200 droplet reader (Bio-Rad) and the data were analyzed by the QX Manager Software 2.1 (Bio-Rad). Reactions were performed in triplicate. The DMR level was normalized against the RPP30 level and then expressed as a fraction of the methylated control.

### Methylated and non-methylated control preparations

To prepare for the swine methylated control, the swine brain gDNA was treated with the CpG methyltransferase (M. SssI, NEB, Ipswich, MA) at 37 °C for 1.5 h. To prepare for the swine non-methylated control, whole genome amplification of the swine brain gDNA was carried out using the REPLI-g kit (Qiagen, Hilden, Germany). DNA was incubated with REPLI-g Mini DNA Polymerase at 30 °C for 16 h. The reaction was inactivated by heating the samples at 65 °C for 3 min. Both methylated and non-methylated DNA samples were purified using the phenol–chloroform method. The quality of methylated and non-methylated DNA samples was checked by MspI and HpaII digestion and the DNA fragments were resolved by 0.8% agarose gel electrophoresis [25].

### MR Imaging and spectroscopy

All animals underwent MR imaging and proton MR spectroscopic imaging (^1^H MRSI) on a 3 T Tim Trio whole-body MR scanner (Siemens, Erlangen, Germany) using a 12-channel phased-array head coil. The anatomical imaging protocol included a 3-plane scout localizer to determine orientation and position of the brain, T1-weighted magnetization prepared rapid acquisition of gradient echo (MPRAGE) imaging and T2-weighted imaging using standard parameters. Additionally, ^1^H MRSI sequence was acquired from each animal. Physiological monitoring including pulse oximetry and vital signs (oxygen saturation and heart rate) was recorded before and during the entire scanning period. All animals were anesthetized with intravenous propofol, intubated, and maintained under anesthesia with isoflurane for MRI experiments.

### Acquisition and analysis of ^1^H MRSI data

Single slice two-dimensional (2D) multivoxel ^1^H MRSI was set up as previously described [27]. Briefly, a point resolved spectroscopy sequence with water suppression by means of a chemical shift selective saturation (CHESS) pulse was employed. The ^1^H MRSI sequence was acquired twice to cover most of the supratentorial brain region. Sequence parameters included: repetition time (TR)/echo time (TE) = 2500/30 ms, number of excitations = 16, field of view = 55 × 55 mm^2^, matrix size = 8 × 8, slice thickness = 7 mm resulting in a voxel size of 6.9 × 6.9 × 7.0 mm^3^, flip angle = 90º and vector size = 1024. The volumes of interest (VOIs) were selected to include injury regions as well as numerous gray-matter and white-matter regions. The scalp, skull base or sinuses were avoided. Outer volume saturation slabs (20 mm thick) were placed outside the VOIs to suppress lipid signals from the scalp. The data sets were obtained using elliptical density-weighted phase-encoding k-space sampling to reduce the acquisition time. Manual shimming was conducted to achieve an optimal full width at half maximum (FWHM) of < 20 Hz of the water signal. A water unsuppressed ^1^H MRSI spectra was also acquired to correct eddy current-induced distortions and for computing metabolite concentrations.

### MRSI analysis

The ^1^H MRSI data were analyzed using a user-independent spectral fit program (Linear Combination (LC) Model) from animals at days 1, 3 and 14 following injuries. The region between 0.2 and 4.0 ppm of the spectrum was analyzed and the following metabolites were evaluated: N-acetylaspartate (NAA, 2.02 ppm), choline (Cho, 3.22 ppm), and Creatine (Cr, 3.02 ppm). The spectral quality of a voxel was assessed by visual inspection and the errors in the spectral fitting routine. The metabolite concentrations from only those voxels were used that had the value of Cramer-Rao lower bounds/standard deviations (SD) less than 20%. The metabolite ratios (NAA/Cr, and Cho/Cr) were computed from multiple gray-matter and white-matter regions covering right and left cerebral hemispheres. The number of voxels (n) encompassing these regions were as follows: cortical regions (n = 4–6); subcortical white matter regions (SCWM; n = 4–6); basal ganglia (BG, n = 2–3); thalamus (n = 3–4); and genu and splenium of corpus callosum (n = 2 in genu and n = 2 in splenium). The metabolite levels in these voxels were averaged for each animal at each time point.

### Statistical analyses

Levels of cfDNA and concentrations of metabolites at different injury levels and times were compared by two-way analysis of variance (ANOVA). If an ANOVA test was found to be significant (*p* < 0.05), a post-hoc test (Bonferroni test) was performed. All data analysis was performed using the statistical tool in GraphPad Software (v10; GraphPad Software, Inc., La Jolla, CA). For the DMR analysis, we utilized the EdgeR package in Seqmonk, with the with SeqMonk’s multiple-testing correction option enabled to adjust p-values for multiple comparisons. A student’s *t* test was used to assess the statistical significance of the ddPCR assays using gDNA samples. CfDNA concentrations, ddPCR levels, and MRS measurements were additionally analyzed using Bayesian mixed effects regression models (Supplementary Method and Data).

## Results

To provide an overview of our findings, we first summarize the key results of this study (Table 1). We show that:

(i) cfDNA methylation profiles capture brain-associated injury signatures, with strong early changes at 6 h post-injury in miCCI, -miRAI, and moRAI, whereas moCCI exhibits more sustained changes across time points.
(ii) Injury-associated DMG patterns differ by type and severity, with miCCI and miRAI linked to cortical and selected subcortical expression contexts, whereas moCCI and moRAI are linked to deeper central brain-region expression contexts.
(iii) Distinct injury types and severities are associated with different biological pathways, including stem cell and inflammatory response (miCCI), growth factor signaling (moCCI), synaptic and neuronal communication pathways (miRAI), and immune signaling pathways (moRAI).
(iv) Brain-associated injury-derived DMRs, including Serine/Threonine Kinase 40 (STK40, miCCI), Factor X (F10, moCCI), Mediator Complex Subunit 27 (MED27, miRAI), and Runt-Related Transcription Factor 1 (RUNX1, moRAI), were validated by ddPCR as biomarker candidates.
(v) ^1^H MRSI analysis indicates that miCCI is associated with reduced choline (suggesting membrane degradation), whereas moCCI, miRAI, and moRAI show increased choline (suggesting membrane turnover). All injury types show reduced NAA, indicating axonal loss.

**Table 1:** Summary of key analyses and observations in swine TBI models

Injury type	miCCI	moCCI	miRAI	moRAI
Methylation Signature	Many moderate changes at 6 h, then decreasing in number	Several moderate changes at all time points	Many large changes at 6 h, then decreasing in number	Several large changes at 6 h, then decreasing in number
Affected Brain Regions	Cortex, midbrain, hippocampus	Hippocampus, midbrain, thalamus	Cortex, hippocampus, midbrain	Hypothalamus, pons, basal ganglia, thalamus
Functional Pathways	Retina morphogenesis, stem cell development and differentiation, inflammatory response	Growth factor response	Synaptic function, nervous system development, cell communication regulation	Immune signaling (IL-2, T-cell, antigen receptor); inflammatory response
Biomarker candidate by ddPCR	STK40	F10	MED27	RUNX1
^1^H MRSI	Cho ↓ in genu, cortex, and SCWM; NAA ↓ in cortex and splenium	Cho ↑ in genu, cortex, and SCWM; NAA ↓ in cortex and splenium	Cho ↑ in genu, splenium, and SCWM; NAA ↓ in cortex	Cho ↑ in genu, splenium, and SCWM; NAA ↓ in cortex
Key Observations	Mild outer cortical trauma with acute cfDNA release, stem cell/inflammatory activation, cell membrane degradation and neuronal loss	Moderate trauma penetrating in deep brain areas with longer lasting cfDNA release, increased cell membrane turnover and axonal loss	Mild shearing force triggering acute cfDNA release, affecting neuronal communication, resulting in increased cell membrane turnover and axonal loss	Immune-driven disruption with acute cfDNA release, affecting deep brain areas, with increased cell membrane turnover and axonal loss

### Swine reference tissues define brain-associated methylation regions for cfDNA analysis

**Fig. 1:**
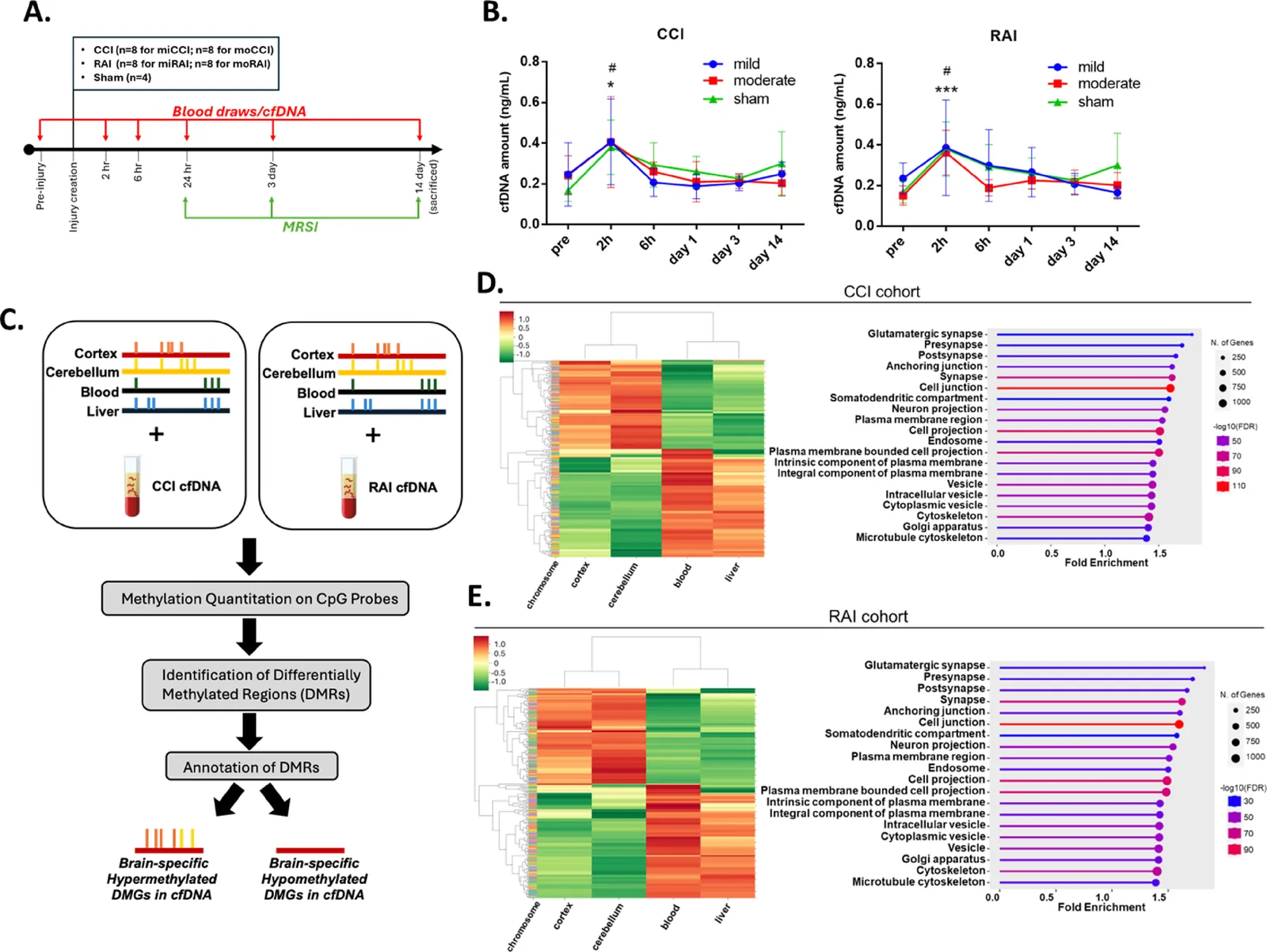
Study workflow identifies brain-associated methylation regions from swine reference tissues. **A** Blood was drawn at pre-injury, 2 h-, 6 h- 3 days-, and 14 days-post injury in the CCI, RAI, and sham groups. MRSI was also performed at 1 day-, 3 days-, and 14 day-post injury. **B** CfDNA was isolated from plasma, and the quantity of cfDNA was determined by Qubit fluorometric quantification. The results are expressed as a fraction of the total plasma volume. Significance was assessed using the two-way ANOVA with Bonferroni’s multiple comparison test. * indicates comparison between the moderate group and pre-injury, while ^#^ indicates comparison between the mild group and sham. *p < 0.05 or ^#^*p* < 0.05; ***p ≤ 0.001. **C** Schematic diagram of methylation analysis. Reference DNA from swine cortex, cerebellum, blood, and liver as well as cfDNA from CCI and RAI were subjected to WGBS. Methylation calls were extracted from.cov files by Bismark and quantitated by the bisulfite methylation over features pipeline in Seqmonk. **D**, **E** Left: Heatmap and dendrogram showing clustering of DMRs with similar methylation values on the side and clustering of tissues on top. CpG probes from the reference tissues were generated with CCI- (**D**) or RAI-derived (**E**) cfDNA, respectively. After methylation quantification. DMRs from the reference tissues were clustered by methylation values and tissue types. The colors in the heat map represent methylation intensity, scaled to mean of zero and unit variance for each DMR. Right: Cellular components in Gene Ontology (GO) enrichment of DMGs identified on the left panel in (**D**) and (**E**)

To elucidate the cfDNA characteristics of the most common types of TBI, we first built two brain injury models in swine, *i.e.* CCI and RAI models, at two injury levels (mild CCI: miCCI; moderate CCI: moCCI; mild RAI: miRAI; moderate RAI: moRAI; Fig. 1A). Blood was collected before the injury (pre-), and 2 h, 6 h, 24 h, 3 days, and 14 days post-injuries (h: hours; d: days). Concurrent ^1^H MRSI was performed at 24 h, 3 days, and 14 days post-injuries to provide independent assessment of the injuries. Next, we examined whether injuries affected the amount of cfDNA released in plasma. Compared to pre-injury, cfDNA levels were significantly elevated at 2 h post-injury in both CCI (*p* < 0.05 for both mild and moderate groups) and RAI models (*p* ≤ 0.001 for the moderate group and *p* < 0.05 for the mild group) (Fig. 1B). CfDNA amounts decreased at 6 h post-injury and continued decreasing in both CCI and RAI models, indicating the surge of cfDNA in plasma was a transient phenomenon in TBI. There was also a non-significant increase in the cfDNA amount at 2 h post sham treatment compared to pre-injury, possibly due to the smaller size of the sham group in our experiment. These data suggest that capturing the elevation cfDNA in TBI is both time sensitive and likely affected by subject variations. Thus, the cfDNA level alone is not an adequate to predict TBI types and severity levels.

Since CCI and RAI represent distinct and fundamentally different types of trauma, we analyzed CCI and RAI brain methylation probes separately using the brain methylation atlas as a reference (see Method, Fig. 1C). Our swine brain methylation atlas includes genomic DNA from the cortex, cerebellum, blood, and liver. Methylation values for CpG probes were quantified and DMRs were identified and annotated to obtain DMGs. We generated CCI- and RAI-based DMRs from CCI- and RAI-derived cfDNA, respectively, along with our swine brain methylation atlas. We found that both CCI- and RAI-based DMRs from both cortex and cerebellum exhibited similar methylation values and clustered more closely together than DMRs from blood and liver (Fig. 1D, E, left panel). This clustering reveals that cfDNA from brain tissues (cerebellum and cortex) exhibit unique and distinct methylation patterns compared to cfDNA from non-brain tissues (blood and liver) upon injury. Functional enrichment analysis using GO for cellular components revealed that brain CCI- and RAI-based DMGs were predominantly associated with synapses, followed by cell junctions, cell/neuron projections, and vesicles releases (Fig. 1D, E, right panel). These functional results further support that brain-associated DMRs are a robust biomarker of TBI.

### CCI produces brain-associated cfDNA methylation changes

**Fig. 2:**
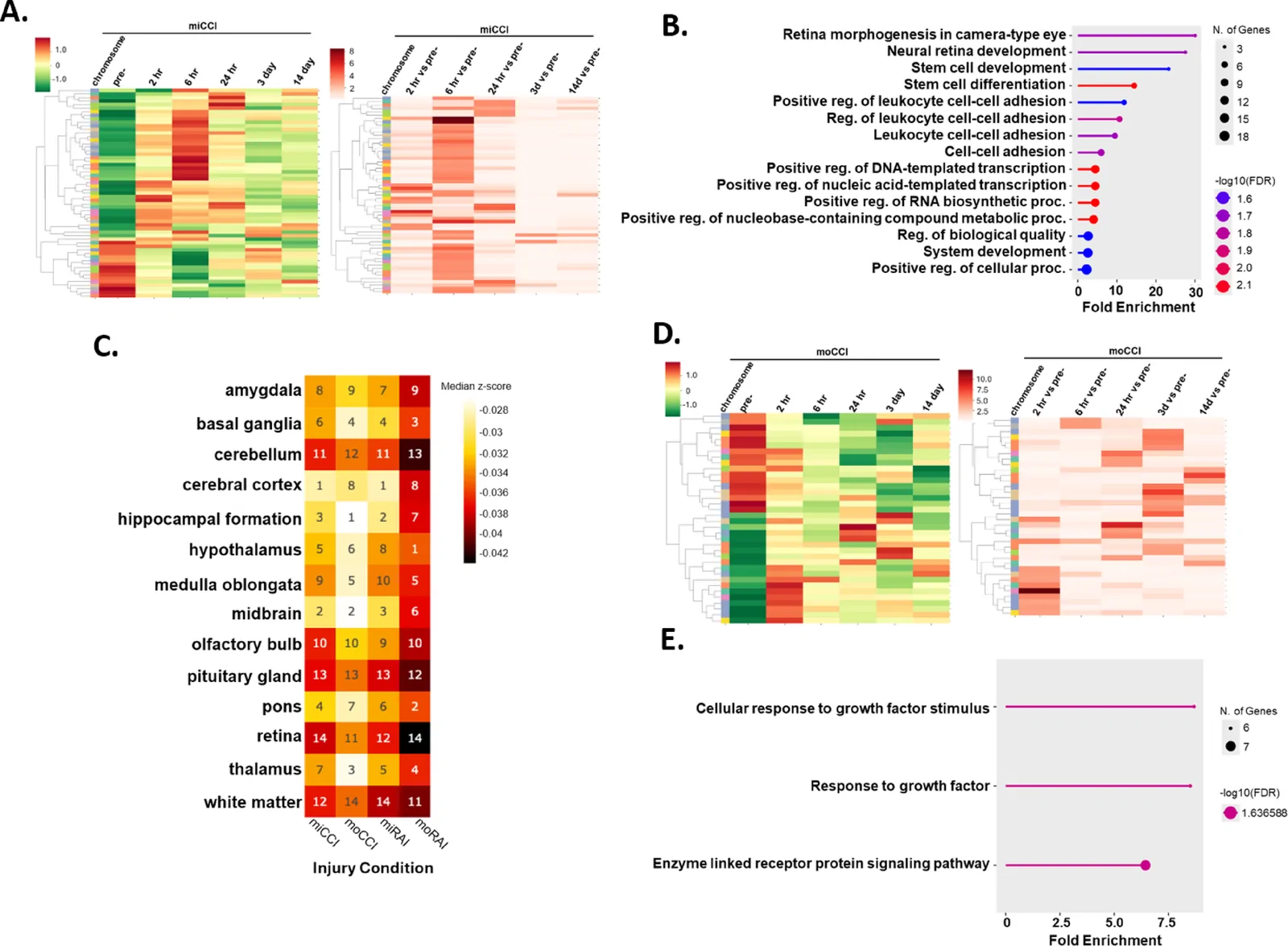
CCI induces brain-associated injury-derived cfDNA methylation signals. **A**, **D** Heatmap of the hierarchical clustering of DMRs from miCCI (**A**) and moCCI (**D**). The dendrogram shows clustering of DMRs with similar methylation values on the side in the indicated cfDNA samples. Left: The colors in the heat map represent methylation intensity, scaled to mean of zero and unit variance for each DMR. Right: The colors in the heatmap represent the negative logarithm (base 10) of the FDR values at the indicated time points. **B**, **E** Biological Process in Gene Ontology (GO) enrichment of brain-associated DMGs in miCCI cfDNA (**B**) and moCCI cfDNA (**E**). **C** Brain-region ranking of DMGs in miCCI, moCCI, miRAI, and moRAI. Heatmap shows HPA pig brain RNA-seq expression-based rankings for each condition using the median RNA expression z-score of the corresponding DMG set. Numbers indicate rank within each condition, with 1 representing the brain region with the highest median expression z-score

We hypothesized that clusters of certain DMRs would exhibit similar dynamic changes after injury. These clusters could be used to both reduce analysis complexity and identify DMRs with consistent and robust temporal changes. To determine which DMRs exhibited similar and different methylation patterns from pre-injury to 14 days post injury, we performed one-dimensional hierarchical clustering of DMRs. We found that brain-associated DMRs in miCCI clustered together based on similar methylation values and diverging methylation patterns from pre-injury to post-injury (Fig. 2A, left panel). This observation suggests that following miCCI injury, different tissue types were releasing cfDNA molecules from pre-injury. We observed significant differences between each post-injury time point and the pre-injury (Fig. 2A, right panel). We identified the percentage of DMRs with significant changes (*p* value < 0.05) and found that highest percentage at 6 h (0.28%), followed by 0.09% at 24 h, 0.06% at 2 h, 0.002% at 3 days, and 0.008% at 14 days post-miCCI. These data indicate that the brain methylation signature of miCCI cfDNA is best captured at the acute phase of trauma, especially at 6 h post- injury. To explore biological pathways associated with methylation changes in DMGs, we conducted functional pathway enrichment of miCCI DMGs using GO biological processes (Fig. 2B). The top identified pathways included retina morphogenesis and development, followed by stem-cell development and differentiation, and leukocyte cell–cell adhesion. To provide brain-region context for the miCCI DMGs, we analyzed HPA pig brain RNA-seq expression patterns for the miCCI DMG set. nTPM values were transformed to z-scores, and brain regions were ranked using the median RNA expression z-score across all miCCI DMGs. The cerebral cortex ranked highest, followed by the midbrain and hippocampus, indicating that miCCI DMGs show higher atlas RNA expression in these brain regions (Fig. 2C). Altogether, miCCI was associated with acute-phase cfDNA release, with injury-associated DMGs showing prominent atlas RNA expression in the cortex and selected central brain regions, consistent with the anatomical context of the CCI model. Moreover, pathway associations related to stem cell development and inflammation may reflect injury-associated repair, developmental, or inflammatory processes.

We also performed similar analysis in moCCI by examining brain-associated DMR clustering in moCCI. Methylation values of the DMRs were more similar across moCCI groups than the pre-injury group (Fig. 2D, left panel). Significant differences (*p* value < 0.05) in methylation values were detected in moCCI and the percentage of DMRs with significant changes were found at highest at 2 h (0.19%), followed by 0.09% at 24 h, 0.08% at 3 days, 0.04% at 14 days, and 0.03% at 6 h post-moCCI (Fig. 2D, right panel). In contrast to miCCI, these methylation changes appeared to spread more evenly across multiple points of observation, suggesting a progressive brain epigenetic signature emerging in blood following moCCI. Next, to evaluate the functional pathways of moCCI DMGs, we conducted GO functional enrichment analysis and identified three biological process pathways (Fig. 2E). The top two pathways were related to the response to growth factor, implicating certain brain recovery may be underway. Last, we found that the highest-ranked brain regions based on median RNA expression z-scores were the hippocampus, midbrain, and thalamus (Fig. 2C). Compared with miCCI, this pattern suggests that moCCI DMGs include genes with stronger expression representation in deeper central brain regions. Together, moCCI cfDNA reflects prolonged trauma over time, with the anatomical context extending to the central brain regions. Additionally, this result indicates that growth factor activation is the primary biological pathway in moCCI.

In sum, these findings suggest that miCCI and moCCI differ in the temporal release of brain-associated cfDNA methylation signatures, with distinct atlas-based brain-region expression patterns and functional pathway associations.

### RAI generates brain-associated cfDNA methylation signatures

**Fig. 3:**
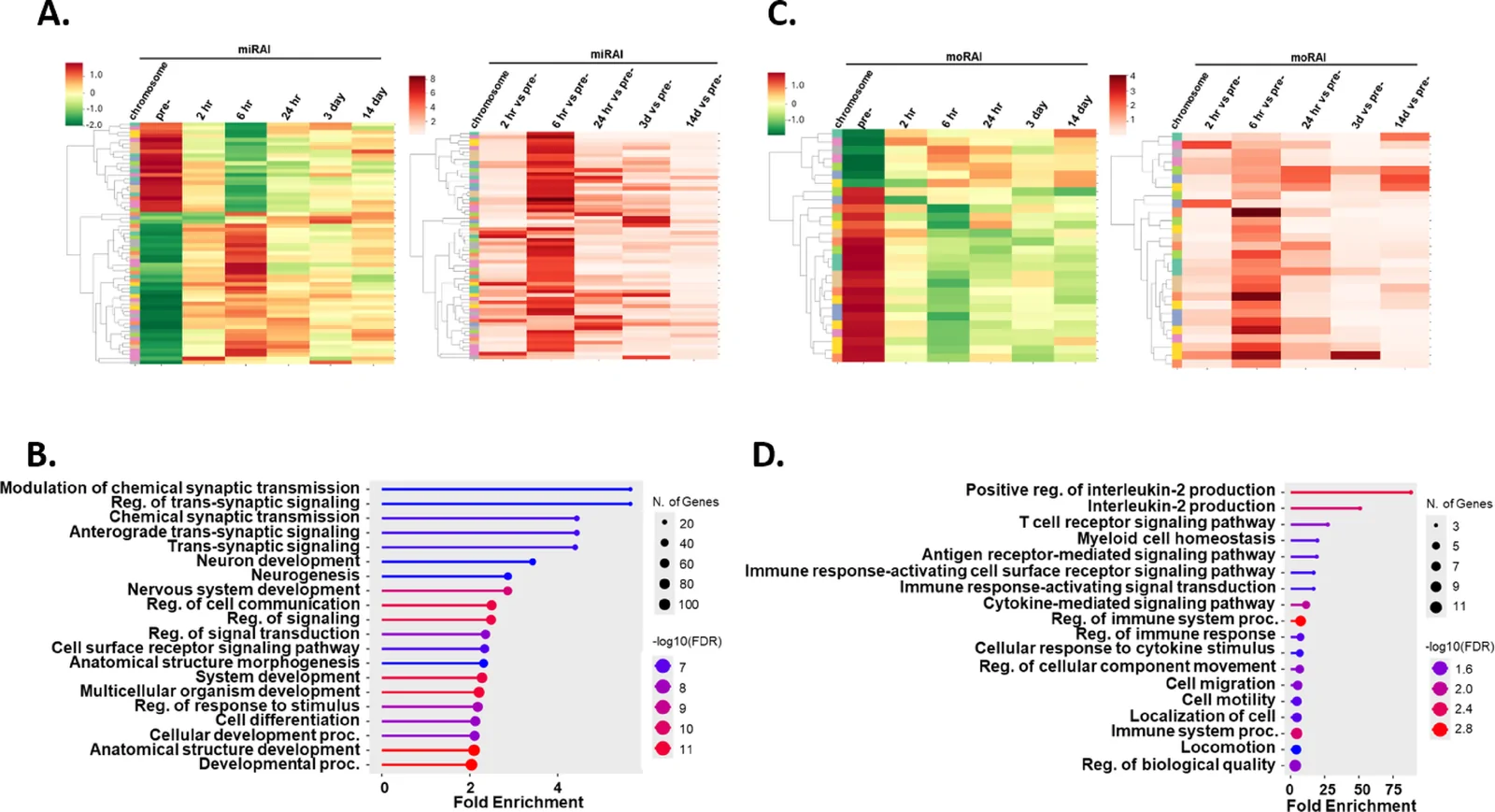
RAI produces brain-associated injury-derived cfDNA methylation changes. **A**, **C** Heatmap of the hierarchical clustering of DMRs from miRAI (**A**) and moRAI (**C**). The dendrogram shows clustering of DMRs with similar methylation values on the side in the indicated cfDNA samples. Left: The colors in the heat map represent methylation intensity, scaled to mean of zero and unit variance for each DMR. Right: The colors in the heatmap represent the negative logarithm (base 10) of the FDR values at the indicated time points. **B**, **D** Biological Process in Gene Ontology (GO) enrichment of brain-associated DMGs in miRAI cfDNA (**B**) and moRAI cfDNA (**D**)

To assess the methylation signature of miRAI, we first examined the brain-associated DMR clustering across the pre-injury condition and the miRAI time points. Similar to miCCI, the methylation values of the DMRs shifted drastically from pre-injury to post-injury time points, particularly at 6 h post-miRAI (Fig. 3A, left panel). The most significant change (*p* value < 0.05) in methylation values was found at 6 h post-miRAI with 3.99% of DMRs identified, followed by 0.66% at 24 h, 0.51% at 3 d, 0.43% at 2 h, and 0.07% at 14d post-miRAI (Fig. 3A, right panel). To assess the functional roles of miRAI DMGs, we performed a GO biological process analysis and identified the top categories. Synaptic pathways were the most prominent, followed by nervous system development and regulation of cell communication (Fig. 3B). The data was supported by GO cellular components which encompassed several ion channels pathways (Supplementary Fig. 1), suggesting that miRAI mostly affects neuronal signal transmission and information integration. Next, to identify brain regions associated with miRAI DMGs, we analyzed HPA pig brain RNA-seq expression patterns for the miRAI DMG set. RNA expression nTPM values were transformed to z-scores, and brain regions were ranked using the median RNA expression z-score across miRAI DMGs. The cerebral cortex ranked highest, followed by the hippocampus and midbrain, indicating that miRAI DMGs show higher atlas RNA expression in regions involved in cortical processing, memory-related circuitry, and central brain function (Fig. 2C). Together, these findings suggest that miRAI is associated with acute-phase cfDNA release, with injury-associated DMGs showing greater atlas RNA expression in the cortex, hippocampus, and midbrain, along with functional associations related to neuronal communication.

Last, to compare the methylation levels of moRAI DMRs before and after trauma, we conducted methylation value clustering across all time points. Methylation values exhibited noticeable changes across all moRAI DMRs compared to pre-injury DMRs (Fig. 3C, left panel). The most significant methylation differences (*p* value < 0.05) were observed at 6 h, with 14.84% of DMRs identified, followed by 3.22% at 24 h, 2.58% at 14d, 1.94% at 2 h, 0.64% at 3 days post-moRAI (Fig. 3C, right panel). To investigate the functional pathways involved in moRAI, we performed GO functional enrichment analysis using moRAI DMGs. The biological process analysis revealed multiple immune signaling pathways, including interleukin-2 production, T cell receptor signaling, and myeloid and antigen receptor pathways (Fig. 3D). Finally, using median expression, we identified the brain regions associated with moRAI DMGs. The highest-ranked regions included the hypothalamus, pons, basal ganglia, and thalamus, indicating that moRAI DMGs show higher atlas RNA expression in deep and central brain regions involved in core neural functions (Fig. 2C). Taken together, our findings suggest that moRAI was associated with the highest acute-phase cfDNA levels, with injury-associated DMGs showing higher atlas RNA expression in central brain regions and pathway associations related to immune processes.

In sum, miRAI and moRAI showed similar temporal patterns of cfDNA release, but differing injury severity was associated with distinct functional pathway signatures and atlas-based brain-region expression patterns.

### Swine injury-associated methylation signatures show human brain enrichment and model-specific cell-type associations

To evaluate whether the swine injury-derived DMGs showed enrichment or genes relevant to human brain biology, we mapped swine CCI/RAI DMGs to their human orthologs and tested whether they were overrepresented among human brain-elevated genes from the Human Protein Atlas. Fisher’s exact test showed significant enrichment of CCI/RAI DMGs in the human brain-elevated gene set (odds ratio = 3.534, p  < × 10^−21^). As specificity control, we performed the same analysis using human blood genes. CCI/RAI DMGs were not enriched in the blood gene set (odds ratio = 0.982, p = 1.000), suggesting that the observed enrichment was not simply driven by blood-associated or hematopoietic genes.

In addition, we performed cell-type association analyses using the top 20 injury-associated DMGs from each injury group (Supplementary Method and Data, Supplementary Fig. 2A–D). In miCCI, the strongest cell-type associations included regulatory T cells and astrocytes. In moCCI, miRAI, and moRAI, the top cell type association was neurons. These findings provide additional support that the swine injury-associated methylation signatures include genes relevant to human brain biology, while also identifying cell-type associations that may reflect both central nervous system and immune-related injury responses.

### ddPCR confirms brain-associated injury-derived cfDNA markers in CCI and RAI

**Fig. 4:**
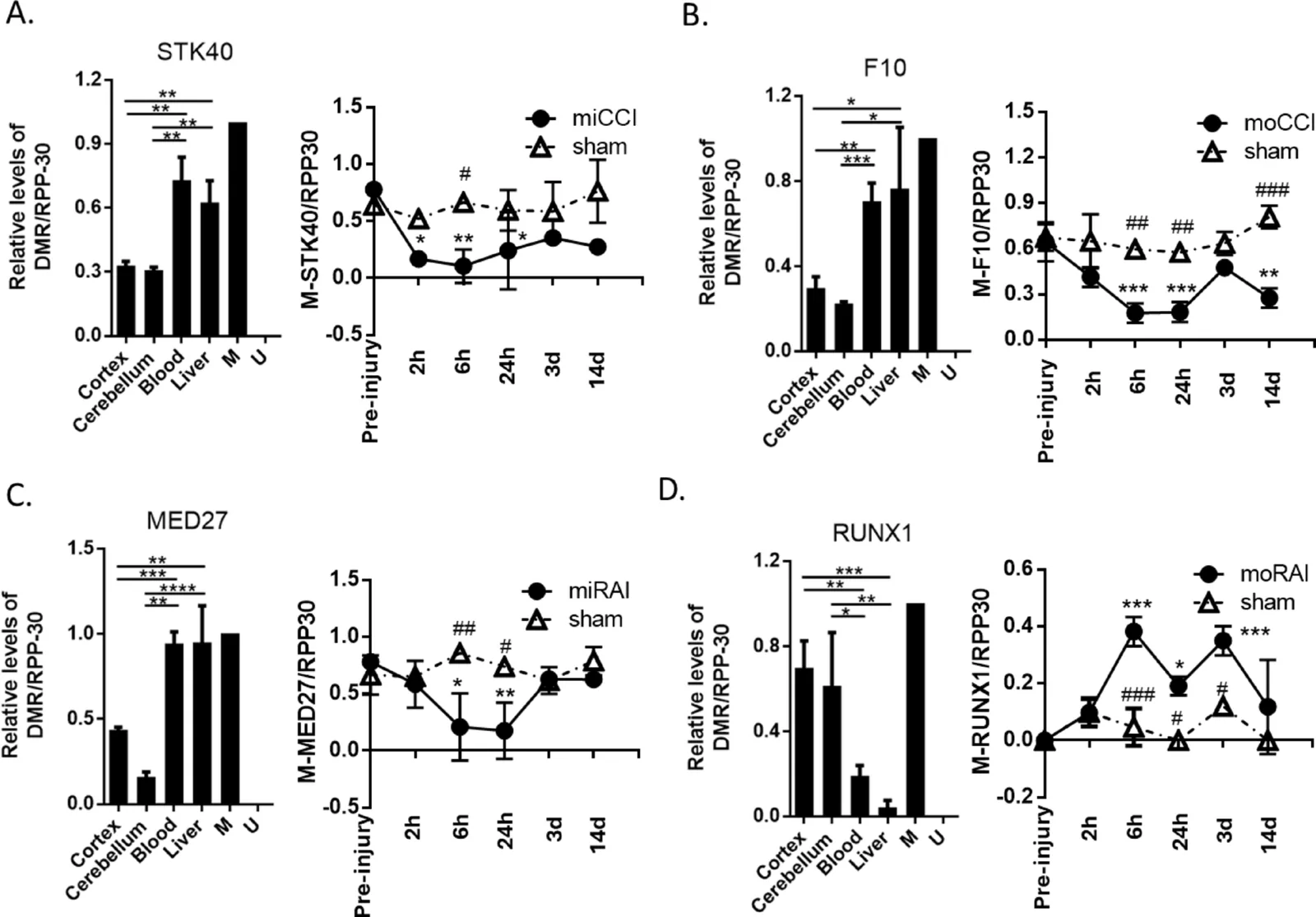
ddPCR confirms injury- and model-specific cfDNA methylation changes in CCI and RAI. **A**–**D** The levels of brain-injury biomarkers were measured using ddPCR probe assays in gDNA (left) and cfDNA (right) samples. DNA from the indicated samples were extracted, bisulfite converted, and subjected to ddPCR analysis. The DMR level was normalized against the RPP30 level and then expressed as a fraction of the methylated control. M: fully methylated control; U: completely unmethylated control. Experiments were conducted in triplicates in gDNA assays and in duplicates from an independent cohort in cfDNA assays. Significance was assessed using the Student’s *t*-test for gDNA assays. *p < 0.05; **p ≤ 0.01; ***p ≤ 0.001; ****p ≤ 0.0001. Significance was assessed using the two-way ANOVA with Bonferroni’s multiple comparison test for cfDNA assays. * indicates comparison to the pre-injury and ^#^ indicates comparison to the sham. *p < 0.05; **p ≤ 0.01 or ^##^*p* ≤ 0.01; ***p ≤ 0.001 or ^###^*p* ≤ 0.001

To develop brain-associated and injury-derived cfDNA as potential trauma biomarkers, we screened the top DMRs with greatest methylation differences between post-injury and pre-injury samples for each injury type using ddPCR (Supplementary Table 1). Both hypermethylated and hypomethylated DMRs (see Method) were included as screening targets and their methylation levels were examined in swine cortex, cerebellum, blood, and liver. Swine fully methylated and non-methylated controls were generated using an in vitro methylation assay and whole genome amplification, respectively (Supplementary Fig. 3A), and were validated by MspI and HpaII digestion (Supplementary Fig. 3B). Among the DMR targets we tested, we identified three hypomethylated DMRs—STK40 in miCCI, F10 in moCCI, MED27 in miRAI—whose methylation levels in the cortex and cerebellum were significantly lower than those in the blood and liver (Fig. 4A–C, left panel; Supplementary Fig. 3C–E). Additionally, we identified one hypermethylated DMR, RUNX1 in moRAI, with methylation levels in the cortex and cerebellum significantly higher than those in the blood and liver (Fig. 4D, left panel; Supplementary Fig. 3F).

Next, we evaluated these injury-derived DMRs using miCCI, moCCI, miRAI, and moRAI cfDNAs. In the miCCI model, STK40 methylation significantly decreased at 2 h, 6 h, and 24 h post-injury compared to pre-injury levels (Fig. 4A, right panel). Additionally, at 6 h post-injury, the methylation level of STK40 in the miCCI group was significantly lower than that in the sham group. In moCCI, the methylation level of F10 significantly decreased at 6 h, 24 h, and 14 days post-injury compared to both pre-injury levels and the sham group (Fig. 4B, right panel). Similarly, in the miRAI model, the methylation level of MED27 was significantly reduced at 6 h and 24 h post-injury compared to both pre-injury levels and the sham group (Fig. 4C, right panel). Last, we detected a significant elevation of methylated RUNX1 at 6 h, 24 h, and 3 days post-moRAI when compared to the pre-injury level and the sham group (Fig. 4D, right panel).

Together, these results demonstrate that brain-associated and injury-derived cfDNA can serve as potential biomarkers for CCI and RAI.

### CCI and RAI produce model-specific changes in choline and NAA metabolism

In addition to cfDNA, brain-associated metabolites characterize the biochemical impact of injury across different brain regions in response to trauma. While NAA levels are often reduced in brain areas affected by neuronal loss or axonal degeneration [28], choline (Cho) levels are usually elevated in injured regions [28, 29] due to cell membrane disruption and turnover [30], Thus, we conducted ^1^H MRS as the non-invasive assessment of these key brain-associated metabolites.

**Fig. 5:**
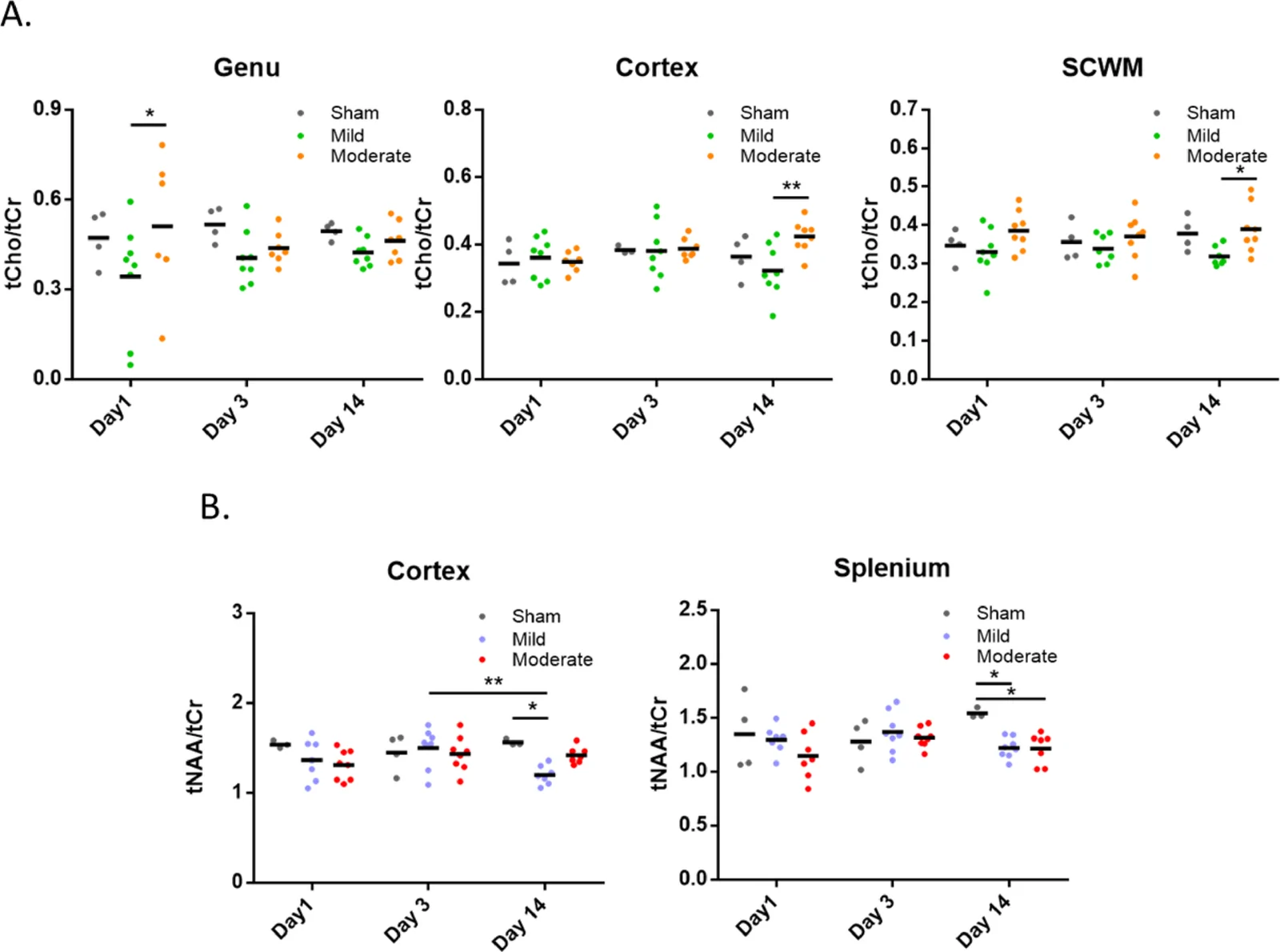
CCI alters choline and NAA metabolite ratios after injury. **A**, **B** The ^1^H MRSI data were analyzed from animals at days 1, 3 and 14 following injury. The metabolite ratios, tCho/tCr (**A**) and tNAA/tCr (**B**), were computed from multiple gray-matter and white-matter regions covering right and left cerebral hemispheres together. tNAA: total NAA; tCr: total creatine; SCWM: subcortical white matter regions. Significance was assessed using the two-way ANOVA with Bonferroni’s multiple comparison test. *p < 0.05; **p ≤ 0.01

To assess the neural damage, we investigated the changes in Cho and NAA levels after CCI and RAI. The gradient echo image for an example slice is shown in Supplementary Fig. 4, with the selected spectra indicating levels of NAA, Cho, and Cr. In CCI, the choline levels at the genu of the corpus callosum (CC) were elevated in moCCI but reduced in miCCI at 1 day post-injury compared to the sham group (Fig. 5A), resulting in a significant difference between moCCI and miCCI (*p* < 0.05). This phenomenon is also observed at 14 days post-CCI in the cortex (*p* < 0.01 between moCCI and miCCI) and SCWM (*p* < 0.05 between moCCI and miCCI) (Fig. 5A), suggesting a distinct difference in injury response biological pathways between moCCI and miCCI. While an increase in choline levels indicates excessive cell injury or death and is commonly detected in TBI [29, 31–34], a decrease in choline levels has also been reported, potentially indicating membrane destruction and glial cell degradation [35]. Compared to the sham group, there was a noticeable, though not statistically significant, decrease in choline levels in the genu of the CC at 3 days and 14 days post-miCCI and -moCCI (Fig. 5A), and in the splenium of the CC at 1 day, 3 days, and 14 days post-miCCI and -moCCI (Supplementary Fig. 5A). Since choline is an important component of phospholipids, our data suggest that a disruption in member turnover plays a role in CCI.

Next, we examined the NAA levels in the above-mentioned brain regions in CCI. In the cortex, NAA levels decreased at 1 days and 14 days post-injury in both miCCI and moCCI compared to the sham group, with a significant reduction observed only at 14 days post-injury in the miCCI group (*p* < 0.05) (Fig. 5B). Additionally, within the miCCI group, NAA levels significantly decreased from 3 to 14 days in the cortex (*p* ≤ 0.01). Similarly, in the splenium of the CC, NAA levels reduced at 1 d and 14 d post-injury in both miCCI and moCCI compared to the sham group, with a significant reduction at 14 d post-injury in both miCCI (*p* < 0.05) and moCCI (*p* < 0.05). While no significant changes were observed in NAA levels in the BG, the genu of the CC, or the SCWM, NAA levels in the genu of the CC consistently decreased at 1 day, 3 days, and 14 days post-injury in both miCCI and moCCI compared to the sham group (Supplementary Fig. 5B). These data are consistent with most other reports indicating that NAA reduction is a common feature in TBI, suggesting that neural damage continued up to 14 days after CCI.

**Fig. 6:**
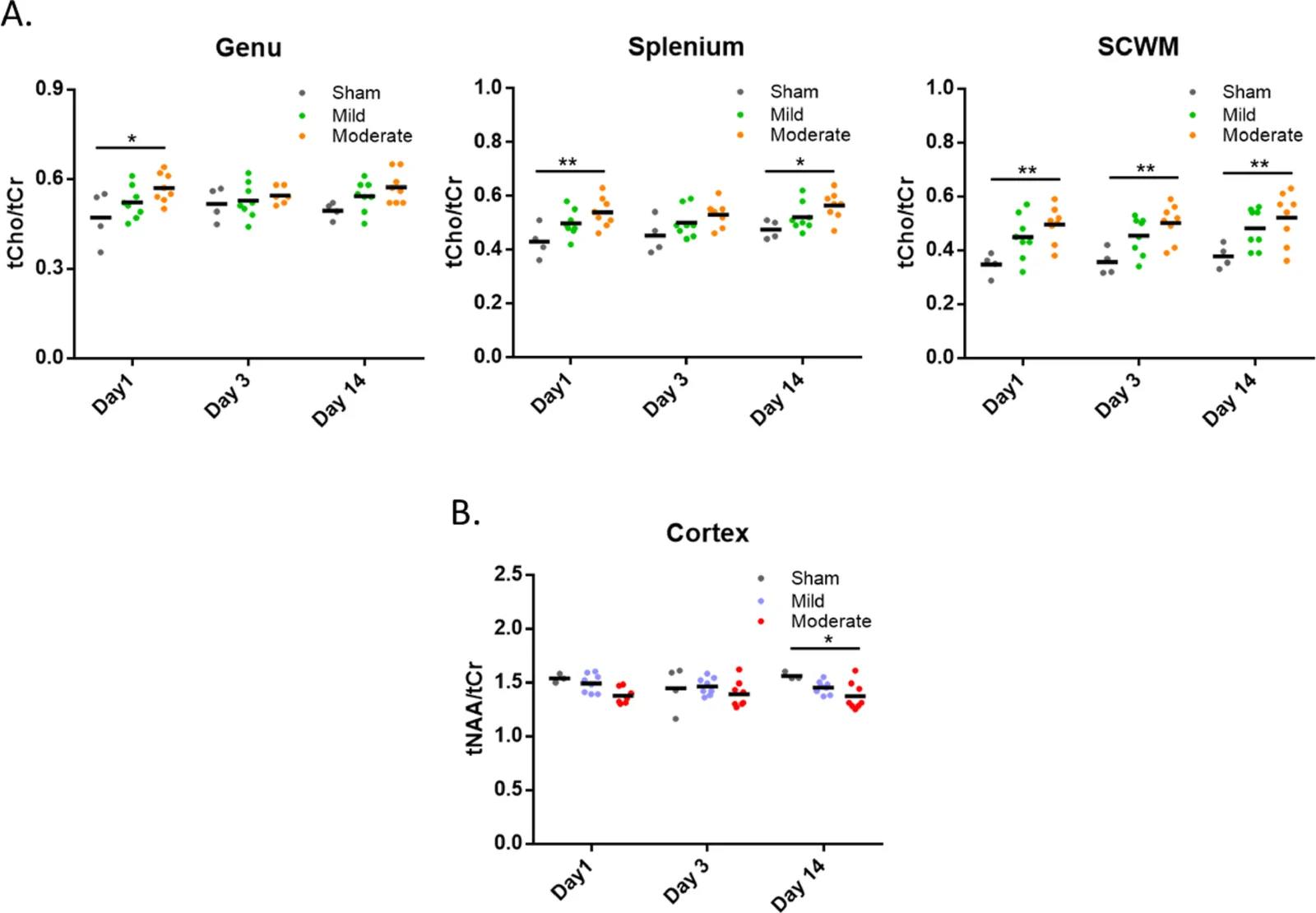
RAI increases choline and reduces NAA metabolite ratios after injury. **A**, **B** The ^1^H MRSI data were analyzed from animals at days 1, 3 and 14 following injury. The metabolite ratios, tCho/tCr (**A**) and tNAA/tCr (**B**), were computed from multiple gray-matter and white-matter regions covering right and left cerebral hemispheres together. tNAA: total NAA; tCr: total creatine; SCWM: subcortical white matter regions. Significance was assessed using the two-way ANOVA with Bonferroni’s multiple comparison test. *p < 0.05; **p ≤ 0.01

To evaluate phospholipid turnover in RAI, we first measured the choline levels and found that they were elevated in most brain regions, including the cortex, the genu and splenium of the CC, and the SCWM (Fig. 6A; Supplementary Fig. 6A). This increase in choline levels appears to correlate with the severity of RAI. A significant elevation in choline levels was found at 1 d post-moRAI in the genu of the CC (*p* < 0.05), at 1 d and 14 d post-moRAI in splenium of CC (*p* < 0.05 at 1 day and *p* ≤ 0.01 at 14 days), and at 1 d, 3 d, and 14 d post-moRAI in SCWM (*p* ≤ 0.01 at 1 day, 3 days, and 14 days), compared to the sham group (Fig. 6B). A reduction in choline levels was seen in miRAI and moRAI compared to the sham group at 3 days post-injury in the BG (*p* < 0.05 in miRAI, Supplementary Fig. 6A). Together, these data demonstrate that the breakdown of phospholipids in RAI, possibly from damaged or dying neuronal and glial cells.

To assess the neural damage in RAI, we evaluated NAA levels and found that the cortex was the only brain region showing major NAA changes (Supplementary Fig. 6B, Fig. 6B). Both miRAI and moRAI showed a decrease in NAA levels in the cortex at 14 d post-injury, with a significant reduction observed at 14 d post-moRAI (*p* < 0.05) (Fig. 6C), indicating that neuronal damage persisted two weeks after the trauma.

While global GO enrichment pathways analyses highlight injury-specific biological processes, selected brain-associated DMGs provide additional context for neurochemical alterations measured by ^1^H MRS. Although these functions are not among the top enriched GO categories, they are represented within brain-associated DMGs and are biologically relevant to MRS-derived measures. For example, *RIN3* is linked to endocytic trafficking, *SCUBE1* and *SLC15A5* are membrane-associated genes, and *NAGA* encodes a lysosomal enzyme that degrades glycoconjugates, which may contribute indirectly to membrane component turnover (Supplementary Table 2). In parallel, genes such as *SEMA6C* and *CPNE5* support neural signaling or synaptic/axon-related biology, whereas *CCDC88C* is involved in regulating neural development. Together, these gene-level observations are consistent with ^1^H MRS findings, supporting membrane disruption and remodeling, as well as altered neural integrity. In sum, these data further demonstrate the utility of combining cfDNA profiling with ^1^H MRS to provide mechanistic insight into brain injury and to aid temporal interpretation of progressive pathophysiology.

## Discussion

In this study, we demonstrate that cfDNA methylation patterns are biomarkers for TBI severity and progression. We present the complete brain-associated methylome profiles for miCCI, moCCI, miRAI, and moRAI. The brain methylome profiling reveals diverse molecular pathways and affected brain regions associated with each injury type and level. We demonstrated that injury-derived DMR levels can be amplified using ddPCR, highlighting their potential use as biomarkers in tracking the progression of brain trauma.

Our functional enrichment analyses, combined with previous IHC results, indicate miCCI triggers stem cell development and inflammation, which is line with other studies where cortical impact stimulates neural stem cell proliferation [36] and leukocytes aggregate in the injured cerebral tissue [37, 38]. This response may represent an endogenous pathway for brain cell regeneration and tissue recovery. Noticeably, our IHC staining data indicates elevated inflammation in miCCI at the perivascular zone, which also houses the neural stem cells and progenitor cells [39], suggesting that the perivascular zone was potentially damaged during miCCI.

In moCCI, functional enrichment pathways suggest that the brain undergoes growth factors-associated processes. It has been shown that nerve growth factor (NGF), a neurotrophin, is excessively upregulated following cortical trauma [40]. While NGF provides neuroprotection [41], proneurotrophins (proNGF) and p75 neurotrophin receptor (p75NTR) can induce apoptotic neuronal death in CCI [42]. Given that most brain-associated cfDNA molecules persist up to 14 days post-moCCI, it suggests that neurorecovery attempts and ongoing brain cell death may coexist over an extended period, especially as the severity of the cortical impact increases.

In our miRAI model, numerous synaptic pathways, including GABAergic and glutamatergic synapses, are enriched. Studies have shown that diffuse structural alterations in the synaptic clefts and postsynaptic densities (PSD) can lead to temporary loss of neural circuit connectivity [43]. Synapse density may decrease for up to a week after TBI, with recovery occurring over approximately 30 days [44]. In the moRAI model, we identified the activation of several immune pathways, including interleukin-2 (IL-2) production, T-cell receptor signaling pathways, myeloid cell homeostasis, cytokine- and antigen receptor-mediated signaling pathways. Neuroinflammation is a common response to TBI. While initial inflammation can support neuron regeneration, excessive or prolonged inflammation after TBI may become neurotoxic, contributing to symptoms such as depression, anxiety, and seizures [45–47]. Interestingly, it has been reported that brain-specific IL-2 production drives Treg cell expansion and enhances neuroprotection following TBI [48]. Since IL-2 production is an enriched pathway in our analysis, it may suggest that the swine brain produces IL-2 as a self-recovery mechanism to modulate inflammation and promote healing after moRAI.

The four ddPCR-validated DMRs mapped to STK40, F10, MED27, and RUNX1. These regions were selected as candidate brain-associated injury markers based on differential methylation in TBI cfDNA and their brain-associated profiles defined using the brain methylation atlas. However, these loci should not be considered brain-exclusive. STK40 is linked to inflammatory regulation [49]; F10 encodes coagulation factor X and has been reported in central nervous system-related contexts [50, 51]; MED27 is a subunit of the Mediator transcriptional complex with roles in neurodevelopment [52, 53]; and RUNX1 is a transcription factor involved in hematopoietic and immune regulation [54]. Prior studies also support RUNX1 expression in neural injury contexts, including injury-associated microglia, neural stem/progenitor cells, and later post-injury neuronal populations [55], In our study, the RUNX1-associated DMR was hypermethylated after injury. Because this DMR was identified as a brain-associated methylation marker and was not linked directly to RUNX1 expression in matched tissue, we interpret this finding as an injury-associated cfDNA methylation change with potential relevance to inflammatory or neural injury responses, rather than as evidence of brain-specific RUNX1 expression or direct transcriptional regulation.

The most prominent metabolite in water-suppressed ^1^H MRSI is NAA, synthesized in neuronal mitochondria from acetyl coenzyme A and L-aspartate by a membrane-bound enzyme [56]. Although its precise function is still not fully known, possible roles include lipogenesis in myelination, ion balance, neuromodulation, and neuronal mitochondria energy metabolism [57]. Because it is almost exclusive to neurons (< 10% contribution from glia and extracellular fluid [58]), NAA is considered as a putative marker of neuronal integrity and viability [59]. While a majority of the studies have demonstrated significant decline in NAA levels following TBI even in brain regions remote from the focal injury [28, 32, 60], a few studies have reported no substantial change in NAA [61, 62]. The reduction in NAA levels have been found to be correlated with severity of injury [31, 63]. Moreover, this reduction in NAA recovered to baseline level in diffuse neuronal injury models at a rate inversely proportional to injury severity and in correlation with brain energy state in a study [64]. In the present study, significant decreases in NAA levels were observed from cortex and splenium of corpus callosum at different post-injury periods in miCCI and moCCI compared to the sham group. Our findings are consistent with previous studies showing decreased levels of NAA from multiple cortical regions [65, 66] and splenium of corpus callosum [67].

Since choline is a metabolic marker of myelin and cellular membrane integrity, alterations in choline levels can reflect various stages of brain injury. Elevated choline levels are often reported during acute and chronic phases of TBI due to neuroinflammation, excitotoxity, ischemia, and oxidative stress [30]. In contrast, decreased choline levels have been reported at the initial stage of trauma as a result of membrane degradation [35, 68, 69]. The total choline levels in CCI show a two-faceted trend. We detected a minor decrease in miCCI and moCCI in some of the brain regions at certain time points. However, as the severity of the impact increased, slight increases in choline levels were noted in moCCI. In contrast, the choline levels show a consistent increase across most brain regions with increasing trauma in RAI. These data suggest that direct cortical impact and rotation head injury involve distinct neurodamage and neurorecovery pathways related to membrane breakdown and degradation.

It is important to distinguish between intracellular methylation dynamics and cfDNA release. DNA methylation changes occur in viable cells as part of stress or injury responses, whereas cfDNA is released following loss of cell integrity [14]. Therefore, the methylation patterns observed in cfDNA likely reflect the epigenetic state of cells prior to their damage or death. These features suggest that cfDNA methylation profiles can serve as a molecular snapshot of injury-associated cellular states and may help inform the evolving pathophysiology of TBI. In addition, circulating cfDNA following TBI may arise from both injured brain cells and peripheral immune cells activated by injury [15, 70]. As cfDNA functions as a damage-associated molecular pattern (DAMP), it may contribute to systemic immune activation and influence the composition of circulating cfDNA [71, 72]. Given the relatively rapid turnover and clearance of cfDNA, the observed methylation profiles likely reflect recent cellular injury and dynamic contributions from both brain and peripheral sources. These factors may partially explain the cfDNA changes observed in both TBI and sham conditions [73, 74].

In this study, cfDNA methylation signatures were validated using ddPCR in a swine TBI model, demonstrating the feasibility of detecting injury-associated epigenetic markers in peripheral blood. From an implementation perspective, while current methylation-based sequencing workflows may require hours to days, targeted approaches such as ddPCR have the potential to reduce turnaround time with further development to meet the rapid time constraints of acute TBI care. Furthermore, by focusing on predefined regions rather than genome-wide sequencing, targeted approaches may mitigate data privacy concerns and improve feasibility for clinical use. Importantly, cfDNA methylation signatures are consistent with MRS findings, where reduced NAA reflects neuronal loss and altered choline indicates membrane turnover, supporting the hypothesis that cfDNA methylation captures biologically relevant injury processes. This suggests a potential role in stratifying TBI subtypes and guiding treatment decisions. For example, biomarkers associated with neuronal or synaptic dysfunction may indicate the need for neuroprotective or rehabilitative strategies. Nevertheless, further work is needed to determine how this information can be integrated into clinical decision-making and translated into targeted therapeutic strategies.

## Limitations

This study is designed as an exploratory, proof-of-concept investigation of plasma cfDNA biomarkers in a swine TBI model. However, several limitations remain. First, the sample size within each injury mechanism and severity is limited, which may introduce variability and affect statistical power. We also observed substantial variability in cfDNA levels among sham subjects, consistent with reports that cfDNA levels can be influenced by biological factors such as age and sex [75, 76]. Second, although CCI [77–79] and RAI [24, 80–82] models have shown to recapitulate key clinical and pathological features of human TBI, this study was conducted in a single, highly controlled large-animal model and may not fully capture the heterogeneity observed in human TBI. As such, these findings require validation in clinically diverse human TBI cohorts to establish generalizability. Third, the functional enrichment pathway analyses are constrained by the current, incomplete knowledge of brain and metabolic pathways. While the analysis identified pathways associated with brain-related processes, these pathways are not necessarily exclusive to the brain, as many relevant mammalian (including human) pathways remain incompletely defined. Finally, although cfDNA levels increased significantly at 2 h after injuries, consistent with previous studies [16, 83], the transient nature of cfDNA elevation suggests that using cfDNA levels alone may have limited utility as a standalone biomarker. In addition, the clinical translation of cfDNA methylation assays remains to be established, including assay turnaround time and feasibility in acute care settings. These limitations highlight the need for further validation in clinically diverse cohorts and continued refinement of cfDNA-based approaches to improve specificity, robustness, and clinical applicability.

## Conclusions

As TBI is complexed by different subtypes and severities, it is important to develop an approach that can provide dynamic and informative insights into brain damage. In this study, we show that cfDNA methylation has the potential to offer lesion site-specific information in a preclinical model, supporting its further investigation as a biomarker for TBI. Moreover, cfDNA technology may complement other diagnostic modalities, such as MRSI and DTI, to enhance TBI characterization. With additional study, cfDNA biomarkers may contribute to improved diagnostics and prognosis in TBI.

## Supplementary Information

Below is the link to the electronic supplementary material.

Supplementary Material 1

Supplementary Material 2

## Data Availability

The WGBS raw data generated in this study have been deposited in the Gene Expression Omnibus (GEO) under the accession numbers GSE304593.

## Abbreviations

BG: Basal ganglia
BP: Biological processes
cfDNA: Cell-free DNA
CC: Cellular components
CVC: Central venous catheter
CHESS: Chemical shift selective saturation
Cho: Choline
CTE: Chronic traumatic encephalopathy
CCI: Controlled cortical impact
Cr: Creatine
CpG: Cytosine-phosphate-guanine
DAMP: Damage-associated molecular pattern
DMG: Differentially methylated gene
DMR: Differentially methylated region
ddPCR: Droplet digital PCR
F10: Factor X
FWHM: Full width at half maximum
GO: Gene ontology
GFAP: Glial fibrillary acidic protein
IL-2: Interleukin-2
MPRAGE: Magnetization prepared rapid acquisition of gradient echo
MED27: Mediator complex subunit 27
M: Fully Methylated control
miCCI: Mild contusional injury
miRAI: Mild rotational injury
moCCI: Moderate contusional injury
moRAI: Moderate rotational injury
NAA: N-acetylaspartate
NGF: Nerve growth factor
NFL: Neurofilament light
nTPM: Normalized transcripts per million
p75NTR: P75 neurotrophin receptor
PSD: Postsynaptic densities
proNGF: Proneurotrophins
^1^H MRSI: Proton magnetic resonance spectroscopic imaging
RAI: Rotational acceleration injury
RUNX1: Runt-related transcription factor 1
STK40: Serine/threonine kinase 40
SD: Standard deviations
SCWM: Subcortical white matter regions
TBI: Traumatic brain injury
2D: Two-dimensional
ANOVA: Two-way analysis of variance
UCH-L1: Ubiquitin C-terminal hydrolase-L1
U: Fully unmethylated (non-methylated) control
VOI: Volumes of interest
WGBS: Whole genome bisulfite sequencing
