# Supplementary material for: Cell-free DNA methylation profiling reveals injury types and severities in swine rotational and contusional models of traumatic brain injury
Source: Acta Neuropathol Commun. 2026 Jul 28;14:199. doi: 10.1186/s40478-026-02384-x (PMC13628837; doi:10.1186/s40478-026-02384-x)
Supplement: Supplementary file 1 [file 40478_2026_2384_MOESM1_ESM.docx]

**Supplementary Method:**

**Differential Methylation Analysis.** Methylation values were quantified using SeqMonk’s “Bisulphite methylation over features” pipeline. The combined value to report was set to *mean*. Positions were included according to the user-defined “minimum count to include position,” and features/windows were retained according to the user-defined “minimum observations to include feature.” Differential methylation was assessed using SeqMonk’s EdgeR *for/rev* statistical filter on replicate sets, with SeqMonk’s multiple-testing correction option enabled. Brain-versus-non-brain reference DMRs were retained using FDR < 0.001, and post-injury versus pre-injury cfDNA DMRs were retained using FDR <0.05. We defined the summary difference as follows:

ΔM_w,A−B_=M_w,A_−M_w,B_

where $M_{w,A}$ and $M_{w,B}$ are the SeqMonk-reported mean methylation values for feature/window $w$ in conditions $A$ and $B$, respectively.

No tissue-of-origin probability or CpG-/DMR-level probability of brain specificity was computed. Brain-associated DMRs were defined operationally using SeqMonk-reported differential methylation results between brain and non-brain reference tissues, followed by FDR-based filtering.

**Cell-type association analysis of top DMGs.** For each injury group, the top 20 CCI and RAI differentially methylated genes (DMGs) were selected based on methylation difference and statistical significance from the brain-associated injury DMR analysis. Swine DMGs were mapped to their corresponding human orthologs. The resulting human orthologs were analyzed using ShinyGO v0.80, which incorporates Azimuth reference datasets, to identify enriched cell-type marker associations. Cell-type associations with an FDR < 0.05 in ShinyGO were considered significant. The degree to which DMGs were associated with specific cell types was ranked first by fold enrichment, followed by FDR and the number of genes.

**Functional Enrichment analysis.** As implemented in ShinyGO, enrichment $P$-values are calculated using the hypergeometric test, and FDR values are computed using the Benjamini–Hochberg method to correct for multiple testing. ShinyGO defines fold enrichment as the percentage of genes in the submitted list that belong to a pathway divided by the corresponding percentage in the background gene set:

$$\text{Fold Enrichment}=\frac{k/n}{K/N}$$

where $n$ is the number of submitted genes, $k$ is the number of submitted genes annotated to a given GO term, $N$ is the number of background genes, and $K$is the number of background genes annotated to that GO term.

**Supplementary Data:**

We performed additional statistical analyses using Bayesian mixed effects regression models to analyze cfDNA, ddPCR, choline and NAA concentrations at different time points and injury types. Bayesian inference models were fit using the *brms* R package and R version 4.6.0. To assess the strength of association between various combinations of fixed effects and interactions, we used the *avg_comparisons* function in the R *marginaleffecs* package. For log-normal distributions we set comparison=”difference”, type=”link”, and transform=exp in avg_comparisons, and highlight only estimates that did not contain 1 in the 95% confidence interval. For ordered beta regression (ddPCR analysis), we set comparison=”difference” and type=”response” and highlight only estimates with a 95% confidence interval that did not contain 0. Specifics on each type of analysis follows.

1) We modeled the cfDNA concentrations using a Bayesian log-normal mixed effects regression model. The model included fixed effects for injury type and time point, with a random intercept for pig ID to account for repeated measurements within subject. Posterior average comparisons were used to estimate differences in expected concentration at each post-baseline time point relative to 0h on the response scale. Estimates are reported as posterior means with 95% credible intervals where the 95% confidence interval does not include 1.0. We found that posterior average comparisons indicated that cfDNA was transiently elevated at 2h with the cfDNA ratio being 0.68 higher at 2h compared to 0h (95% CI = [0.37, 1.05]).

2) DdPCR concentration values were bounded on the unit interval [0,1]. Therefore, we modeled the ddPCR concentrations with an ordered beta regression model fit using the ordbetareg R package, which is a front end to brms used in other analyses. The fixed effects models in this model were injury type and time point, with a random intercept for pig ID to account for repeated measurements within animals. Time points were treated as a categorical factor with 0h being the reference. Posterior estimates were reported as posterior means with 95% confidence intervals. Model convergence was assessed using standard Bayesian diagnostics, including visual inspection of trace plots, effective sample and Rhat values.

We found for RUNX1, compared to 0h, ddPCR predicted concentrations tended to increase by 0.227 at 6h (95% CI = [0.083, 0.455]), by 0.093 at 1d (95% CI = [0.003, 0.278]), and by 0.223 at 3d (95% CI = [0.086, 0.450]). Increases at other time points were positive but less certain since their 95% CI included 0. These results suggest a transient increase in RUNX1, peaking 6h and 3d.

For STK40, we found a transient decrease with predicted concentrations decreasing by -0.356 (95% CI = [-0.574, -0.108]), 0.274 (95% CI = [-0.511, -0.031]), and 0.240 (95% CI = [-0.466, -0.011]) at 2h, 6h, and 3d, respectively.

Finally, for F10 the strongest evidence was a transient decrease with predicted concentrations decreasing by -0.277 (95% CI = [-0.456, -0.089]) at 6h, and -0.280 (95% CI = [-0.456, -0.089]) at 1d.

3) Both choline and NAA metabolite concentrations are greater than zero, and right-skewed and can be modeled well with a log-normal distribution. We performed an inverse log transformation on contrasts from the log-normal model and obtained estimates from the model represented ratios of choline concentration with values below one indicating lower choline. Fixed effects were injury group, brain region, and time point. We also include all two-way interactions among these factors. A random intercept model for pig ID was included for repeated measures. The model can be written as:

$$log(\text{choline/NAA})\sim Normal(\mu,\sigma)$$

Individual results are as follows.

**miCCI vs sham**

We found choline ratios of 0.689, 0.732, and 0.742 at days 1, 3, and 14, respectively. These values correspond to approximately 25-31% reduction in choline in miCCI compared to sham. The 95% confidence intervals for genu did not include 1.0, and hence the strongest evidence for changes to choline in miCCI compared to sham occurred in genu.

We found that NAA ratios were at 0.739, 0.769, and 0.685 at days 1, 3, and 14, respectively, corresponding to approximately 25-32% reduction in genu in miCCI compared to sham. The results are congruent with that of choline. In addition, NAA ratio was also 0.836 in cortex on day 14. These results represent the strongest evidence for changes in NAA in the genu region.

**moCCI vs sham**

Choline ratios in basal ganglia (BG), cortex, and thalamus at day 1 were approximately 39%, 30%, and 33% higher, respectively in moCCI compared to sham.

Compared to sham, the strongest evidence for a decrease in NAA was a predicted 20% (95% CI = [0.669, 0.949] decrease in NAA in genu and a predicted 16% (95% CI = [0.707, 0.993]) decrease in thalamus.

**miRAI vs sham**

Choline ratios in SCWM at days 1 and 14 were approximately 29% and 28% higher, respectively in miRAI compared to sham.

**moRAI vs sham**

Choline ratios in cortex at day 1 was approximately 26 % higher, and in SCWM at days 1, 3 and 14 were approximately 43%, 38%, and 39% higher in moRAI compared to sham.

**Interactions: Injury x Time**

**miCCI vs sham**

At days 1,3, and 14 the miCCI/sham NAA ratio in genu was estimated to be 23% lower compared to BG. Thus, the relative miCCI choline increase was much higher in SCWM compared to BG.

**moCCI vs sham**

In all brain regions, the moCCI/sham ratio on days 3 and 14 was estimated to be approximately 0.79 times the moCCI/sham ratio on day 1. This result suggests that moCCI-associated increases were highest on day 1.

**Injury x Region**

**miRAI x sham**

At days 1,3, and 14 the miRAI/sham ratio in SCWM was estimated to be 35% higher compared to BG. Thus, the relative miRAI choline increase was much higher in SCWM compared to BG.

**moRAI x sham**

At days 1,3, and 14 the moRAI/sham ratio in SCWM was estimated to be 41% higher compared to BG. Thus, the relative moRAI choline increase was much higher in SCWM compared to BG.
