# Supplementary material for: Cell-free DNA methylation profiling reveals injury types and severities in swine rotational and contusional models of traumatic brain injury
Source: Acta Neuropathol Commun. 2026 Jul 28;14:199. doi: 10.1186/s40478-026-02384-x (PMC13628837; doi:10.1186/s40478-026-02384-x)
Supplement: Supplementary file 2 [file 40478_2026_2384_MOESM2_ESM.docx]

Supplementary Files

**Supplementary Figure 1. CCI and RAI induce brain-associated injury-derived cfDNA methylation signals. (A)** The total number of brain-associated DMRs in miCCI and moCCI (left) and the breakdown number of hypermethylated and hypomethylated DMRs in miCCI and moCCI (right). (**B**) The total number of brain-associated DMRs in miRAI and moRAI (left) and the breakdown number of hypermethylated and hypomethylated DMRs in miRAI and moRAI (right). (**C**) Cellular component in Gene Ontology (GO) enrichment of brain-associated DMGs in miRAI cfDNA.

**Supplementary Figure 2. Cell-type analysis of the top 20 CCI and RAI DMGs identifies neuronal, astrocytic, and immune-related associations. (A–D)** The top 20 DMGs from each injury group were mapped to human orthologs and cross-referenced with Azimuth reference datasets to evaluate cell-type associations of injury-associated methylation changes

**Supplementary Figure 3. Methylated and unmethylated swine genomic DNA controls support ddPCR testing of candidate injury biomarkers in CCI and RAI. (A)** Workflow of the preparation of methylated and non-methylated standards from swine brain genomic DNA (gDNA). The methylated standard was generated by the *in vitro* methylation assay using CpG methyltransferase, M.SssI. The non-methylated standard was prepared by whole genome amplification using the REPLI-g mini kit. (**B**) MspI (CpG methylation insensitive) digested both methylated and non-methylated DNAs whereas HpaII (CpG methylation sensitive) only digested non-methylated DNA, as resolved by 1% gel agarose. (**C-F**) Selective brain-associated biomarkers were tested using the EvaGreen assay by ddPCR in swine miCCI (**C**), moCCI (**D**), miRAI (**E**), and moRAI (**F**) cfDNA samples. The DMR level was normalized against the RPP30 level and then expressed as a fraction of the methylated control. Experiments were conducted in triplicates. Significance was assessed using the Student's t-test for gDNA assays. *p<0.05.

**Supplementary Figure 4. Representative spectra illustrate choline/creatine and N-acetylaspartate/creatine metabolite quantification.**

**Supplementary Figure 5. Choline and NAA metabolite ratios across CCI groups measured by MRSI. (A-B)** The ^1^H MRSI data were analyzed from animals at days 1, 3 and 14 following injury. The metabolite ratios, tCho/tCr (A) and tNAA/tCr (B), were computed from multiple gray-matter and white-matter regions covering right and left cerebral hemispheres together. tNAA: total NAA; tCr: total creatine; SCWM: subcortical white matter regions.

**Supplementary Figure 6. Choline and NAA metabolite ratios across RAI groups measured by MRSI. (A-B)** The ^1^H MRSI data were analyzed from animals at days 1, 3 and 14 following injury. The metabolite ratios, tCho/tCr (A) and tNAA/tCr (B), were computed from multiple gray-matter and white-matter regions covering right and left cerebral hemispheres together. tNAA: total NAA; tCr: total creatine; SCWM: subcortical white matter regions. Significance was assessed using the two-way ANOVA with Bonferroni's multiple comparison test. *p<0.05.

Table S1. Sequence of the oligonucleotides for ddPCR

Table S2. Selected brain-associated DMGs linked to MRS interpretation
